# Enhancing Buttock Contours: A Safer Approach to Gluteal Augmentation with Ultrasonic Liposuction, Submuscular Implants, and Ultrasound-Guided Fat Grafting

**DOI:** 10.3390/jcm13102856

**Published:** 2024-05-12

**Authors:** Ahmed Elsaftawy, Patryk Ostrowski, Michał Bonczar, Mateusz Stolarski, Kamil Gabryszuk, Tomasz Bonczar

**Affiliations:** 1 Chiroplastica—Lower Silesian Centre of Hand and Aesthetic Surgery, 54-117 Wroclaw, Poland; 2 Department of Anatomy, Jagiellonian University Medical College Cracow, 33-332 Cracow, Poland; 3 Youthoria, Youth Research Organization, 30-363 Cracow, Poland

**Keywords:** gluteal augmentation, ultrasonic liposuction, submuscular implants, fat grafts, surgery

## Abstract

**Background**: The global popularity of gluteal augmentation has risen significantly, driven by the desire for enhanced buttocks size and shape to align with individual patient preferences. This increased demand has prompted extensive research into diverse techniques and their safety. **Methods**: A retrospective analysis was conducted to evaluate the outcomes of a gluteal augmentation technique involving ultrasound-assisted liposuction, submuscular implants, and ultrasound-guided fat grafting. Our study involved a review of the medical records of 50 consecutive patients who underwent this procedure between February 2020 and July 2023. **Results**: Data related to patient demographics, the quantity of fat grafts, and any complications were analyzed. Additionally, a brief survey was conducted to evaluate patient satisfaction. The Polytech implants were used in forty-four patients, and Sebbin implants in six. The implant size varied from 285 to 560. Most of the Polytech implants were 390 cc (25/44; 56.8%). Two patients had a minor infection at the incision sites and subsequent wound dehiscence. No revision surgeries were needed. **Conclusions**: The presented technique incorporates ultrasonic liposuction, submuscular silicone implants, and ultrasound-guided fat grafting to achieve safe and aesthetic gluteal enhancements. This method is especially suitable for patients looking to augment both the central and lateral areas of the buttocks, particularly when they lack adequate fat tissue for augmentation through fat grafting. With the addition of ultrasound guidance, the fat grafting step is significantly safer.

## 1. Introduction

Gluteal augmentation has surged in global popularity as it addresses the desire for enhanced buttocks size and shape to meet individual patient preferences [1]. This heightened demand has led to extensive research into various techniques and their safety [2]. Implants offer a favorable option for patients lacking sufficient adipose tissue for fat grafting. Gluteal augmentation with implants involves variations in implant types and placement planes, each presenting distinct advantages and disadvantages, such as implant protrusion and palpability issues in the lateral gluteal region [3,4,5].

Conversely, individuals with larger amounts of fat may choose augmentation through liposuction and fat grafting, a procedure with numerous protocol variations at each stage. Similar to implant-based techniques, these variations have unique pros and cons [6]. A significant stage of variation in the procedural protocol, with immense importance for patient safety, revolves around the method of fat injection, which is performed either subcutaneously or intramuscularly [6]. However, recent safety recommendations have stressed the importance of performing only subcutaneous injections using intraoperative ultrasound imaging due to the heightened risk of life-threatening complications associated with intramuscular administrations [7,8,9,10].

Combining both of the aforementioned techniques presents a promising solution, aiming to achieve the desired buttocks projection associated with implants while minimizing palpability and protrusion issues, particularly in the lateral gluteal region. Moreover, supplemental fat graft injections to the sides of the buttocks can increase the hip-to-waist ratio, a frequent goal of patients seeking to enhance their buttocks size. This approach particularly benefits patients with limited body fat for gluteal augmentation with liposuctioning and fat grafting. Previous studies demonstrating similar techniques combining implants with fat grafting have been presented in the literature [11]. However, the fat grafting step in those papers was not performed under imaging guidance, an action that may increase the risk of potentially fatal complications [8]. The present study introduces a safer approach to gluteal augmentation involving ultrasonic liposuction, silicone implant insertion into the submuscular plane, and ultrasound-guided fat grafting performed on a cohort of 50 patients.

## 2. Materials and Methods

We conducted a retrospective analysis to evaluate the outcomes of a novel gluteal augmentation technique involving ultrasound-assisted liposuction, submuscular implants, and fat grafting. Our study involved a review of the medical records of 50 consecutive patients who underwent this procedure between February 2020 and July 2023. The present study strictly followed the ethical guidelines outlined in the Helsinki Protocol, upholding principles such as autonomy, beneficence, and non-maleficence. To maintain the highest patient confidentiality and anonymity standards, we exclusively relied on retrospective data from medical records. All patients provided informed consent for the surgical procedure and their participation in the study.

All of the patients underwent an inclusion/exclusion process. The inclusion criteria included (1) gluteal ptosis and/or gluteal flaccidity, (2) gluteal skin that does not reflect pathological changes, (3) no significant comorbidities, (4) a too-low body fat percentage for satisfactory gluteal augmentation with liposuction and fat grafting, (5) a thin patient wanting a larger implant than indicated, and (6) a patient wanting the gluteal projection associated with implants and a larger hip-to-waist ratio. All patients who did not meet the inclusion criteria were excluded from surgery and, consequently, from the present study. Furthermore, additional exclusion criteria were applied, including (1) chronic gluteal pain, (2) spinal disk herniation, and (3) enough body fat to meet the expectations of the patient for buttocks augmentation with fat grafting. Patients who were smokers were advised to quit smoking at least six weeks before the surgery and at least six weeks after.

**Figure 1:**
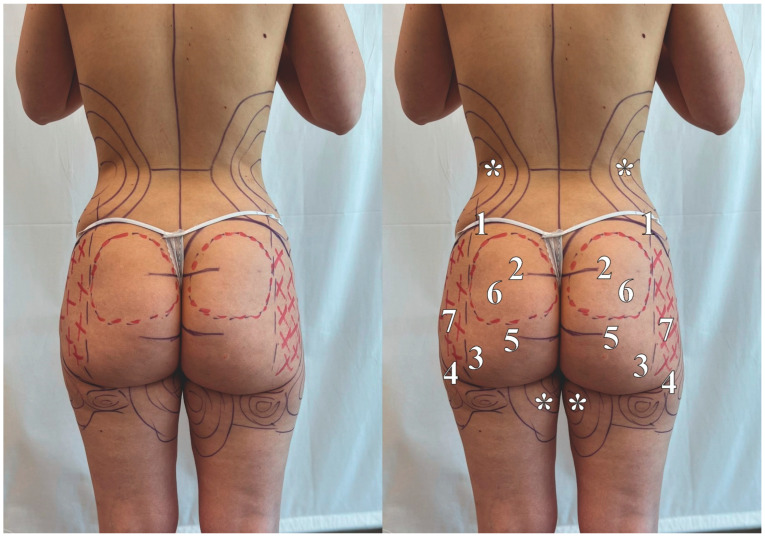
Preoperative markings: (1) posterior iliac crest; (2) horizontal line showcasing the superior limit of the intergluteal fold; (3) vertical line at the level of the greater trochanter (lateral limit of dissection); (4) horizontal line at the subgluteal crease; (5) horizontal line at the level of the coccyx (inferior limit of dissection); (6) location of the silicone implant; (7) area for fat graft injection; * areas for liposuctioning.

Preoperative markings were performed to visualize the areas for liposuctioning and fat grafting and to demarcate the incisions and borders of dissection during the submuscular implant-based technique. The preoperative markings are shown in Figure 1. The most common areas for fat harvesting included the posterior flanks, the lower back, and the upper posterior thighs. The incisions for the ultrasonic liposuctioning were marked in the following locations: bilateral markings superiorly to the superior posterior iliac spine, a singular incision at the upper apex of the intergluteal cleft, and bilateral markings at the midpoint of the subgluteal cleft. The preoperative markings for the submuscular implant-based operation have been described by us in the past [5]. We marked the inferior dissection limit as a horizontal line running from the tip of the coccyx to the greater trochanters. This line is important, as various crucial neurovascular structures, including the sciatic nerve, pass inferior to it. The lateral limit was determined by the posterior border of the trochanter. Next, the medial limit was defined by the ischial tuberosity. The implant size and placement were determined with the aforementioned limits. Moreover, further markings were made to reduce the potential of upper fullness. These included simple markings between the posterior iliac crest and the abovementioned horizontal line connecting the tip of the coccyx to the greater trochanter (referred to as Line A), as well as between the horizontal line and the subgluteal crease (referred to as Line B). To prevent the risk of excessive elongation, shortening of the gluteal region, and disproportionate upper fullness, it has been stated that Line A should be at least double the length of Line B [12]. Finally, the location of the incisions for the implant pocket dissection was marked bilaterally in the intergluteal region. The incision lines were marked on the left and right, 1 cm from each other. The lines were 3 cm long, parallel to each other; however, they were not at the same height (the left incision was higher than the right). The difference in height makes it easier for right-handed surgeons to dissect the pocket of the implant at the appropriate level and vice versa for left-handed surgeons.

All of the operations were performed using general anesthesia. During the perioperative period, a prophylactic antibiotic regimen was utilized. It consisted of administering 2 g of intravenous ceftriaxone. This proactive antibiotic use primarily aimed to prevent infections caused by Staphylococcus aureus and Streptococci strains.

The surgery began with ultrasonic liposuction of the demarcated areas for fat harvesting (Supplementary Video S1 demonstrates our technique). First, injection of an infiltration solution consisting of 1 L of 0.9% saline, 20 mL of 2% lignocaine, 500 mg of tranexamic acid, and epinephrine at a concentration of 1:500,000 was performed. This solution facilitates fat infiltration, enhances the transmission of ultrasonic waves into the fat, reduces the risk of heat-induced injuries, and minimizes blood loss. Additionally, 2% lignocaine may contribute to effective immediate postoperative pain management.

Ultrasonic liposuction was conducted using a VASER device (Sound Surgical Technologies, Louisville, CO, USA). This device effectively disintegrates adipose tissue prior to suctioning. The ultrasonic waves were transmitted continuously, with the wave amplitude set between 70% and 80%. The liposuctioning was performed with a crisscross technique towards the location of the other port incision to ensure symmetrical and aesthetic outcomes. From the lower back and the lower flanks, mostly deep adipose tissue and some subcutaneous adipose tissue were broken down, while only deep adipose tissue was broken down in the lower thighs. Finally, the fat was harvested with a 3.5 mm cannula.

The harvested fat was placed in a sterile, sealed container and settled for 30 min, separating the adipose cells from the liquid. Afterward, the liquid was removed, leaving only the fat in the container. The fat was then drawn into 60 mL syringes and centrifugated to separate any remaining fluid from the fat. In addition, venous blood was drawn from the patient to create platelet-rich plasma. Finally, the isolated fat was combined with the platelet-rich plasma in a 1:5 ratio, primarily to enhance the fat graft’s survival, as stated in the literature [13]. Once these steps were completed, the fat graft was ready for transfer to the gluteal region.

Before the injection of the fat graft, the insertion of the silicone implants into the submuscular plane was performed. We have demonstrated this technique in a previous paper [5], which was based on the one described by Petit et al. [14]. The instruments used in this surgical technique included a Keller-funnel 2 (Allergan, Inc., Dublin, Ireland), curved-tip Mayo scissors, a Collin Hartmann retractor, and two spacers, one with a heart-shaped tip and one with a round tip. Moreover, silicone smooth surface implants with round bases were used. The implants were the Sebbin round gluteal implants (Sebbin, Paris, France) and Polytech Round POLY smooth implants (Polytech, Dieburg, Germany). The procedure began by injecting the aforementioned incision lines with a solution consisting of 2 mg of epinephrine diluted in 20 mL of ropivacaine 7.5 mg/mL in 1000 mL of saline with a 22-gauge needle. Next, stab incisions were made along both incision markings with a 15-scalpel blade. Subsequently, the solution was injected into the submuscular layer using a 15 cm long, 2.5 mm smooth infiltration cannula with the same volume on each side of the buttocks. The surgical technique used in the present study has been described in the literature [5,14]. It started by making an incision on the skin using a 15-scalpel blade on the marked incision lines and dissecting the area until the lateral border of the sacrum was reached. Next, perforation of the gluteal maximus fascia was performed using the closed tip of the scissors, making a small hole. This hole was subsequently enlarged by opening up the scissors, which allowed the surgeon to insert his index finger into the submuscular area. A small cavity was created with blunt dissection, and long spacers were inserted to enlarge this area on the caudal, cranial, and lateral edges and free it from strong fascial adhesions. To maintain the size of the cavity and keep it dry while dissecting the other side, a laparotomic gauze was inserted into the pocket. Finally, the silicone implants were inserted into the pocket, covered by the gluteus maximus muscle.

The final step of our novel technique was the injection of the fat graft. It is extremely important to perform the injection process only in the subcutaneous layer and not intramuscularly due to the increased risk of life-threatening complications associated with the latter [15]. Therefore, the injection process was performed with intraoperative ultrasound imaging. A 4.0 mm blunt cannula was used to inject the fat graft. The volume of the fat graft varied between 300 and 350 mL. The injection process was performed with a continuous motion to avoid accidental injection into blood vessels and to ensure that the fat graft was evenly distributed. The fat graft went mainly to the lateral buttocks, as marked in Figure 1. This, combined with ultrasonic liposuction on the lower flanks and back, helped achieve the desired hip-to-waist ratio by reducing waist width and increasing hip width. Furthermore, the fat graft may also reduce any potential implant palpability or visibility in the lateral edges of the buttocks.

The pain management plan included paracetamol (4 doses of 500 mg each) and ketoprofen (2 mg twice daily) for pain relief, along with diazepam (2 mg) as a muscle relaxant. In cases of more severe pain, tramadol was prescribed. Moreover, Enoxaparin 40 mg was administered as an anticoagulant treatment for a duration of 10 days. Patients were advised to avoid strenuous physical activities for four weeks following the surgery. The initial postoperative checkup occurred seven days after the surgery, during which the wound dressings were removed, and incisions were cleaned with saline solution. Patients were also encouraged to wear compression garments for at least three weeks post-surgery to aid in buttock shaping. Additionally, lymphatic massages were integrated into the postoperative care plan, starting five to seven days after the surgery and continuing with two weekly sessions for five weeks. Afterward, patients had follow-up appointments at six weeks, twelve weeks, and six months.

## 3. Results

A retrospective analysis of the results of 50 female patients who underwent a gluteal augmentation was performed. The age of the patients ranged from 21 to 52 years, with a mean age of 33.90 years old (SD = 6.83). The weight of the patients ranged from 47.50 to 101.00 kg, with a mean weight of 59.23 kg (SD = 8.52). The analyzed patient characteristics are presented in Table 1. The satisfaction of the patients was assessed with a short survey, and the results are presented in Table 2.

**Figure 2:**
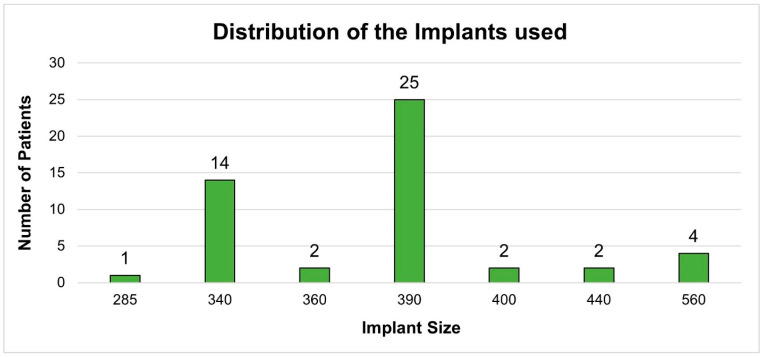
Distribution of the implants used in the performed surgeries.

The Polytech implants were used in forty-four patients, and Sebbin implants in six. The implant size varied from 285 to 560. Most of the Polytech implants were 390 cc (25/44; 56.8%). The Sebbin implants included 360 cc, 400 cc, and 440 cc; two of each were used (2/6; 33.3%). The data regarding the implants used can be found in Table 3 and Figure 2. Three of our patients had complications. Two patients had a minor infection at the incision sites and subsequent wound dehiscence. The wound dehiscence was relatively small without the presence of implant protrusion. The affected incision sites were cleaned with saline and treated with intravenous broad-spectrum antibiotics, mainly amoxicillin, 400 mg two times a day, and metronidazole, 500 mg twice daily for five days. One patient had sciatic neuropathy; however, it resolved after three weeks. Most importantly, no revision surgeries were needed. Moreover, no cases of hematomas or seromas were noted.

## 4. Discussion

Our approach to gluteal augmentation provided reliable and satisfactory aesthetic outcomes while maintaining a high level of safety for patients who were unsuitable for buttocks augmentation via liposuction and fat grafting or isolated implant-based buttocks contour enhancement. This study is the first to demonstrate the combination of ultrasonic liposuction, submuscular implant insertion, and ultrasound-guided fat grafting. One of the primary advantages of ultrasonic liposuction is its precision and selectivity in targeting fat cells. Through the application of ultrasound energy, this method can effectively break down fat cells while preserving adjacent structures such as nerves, blood vessels, and connective tissue. This precision substantially reduces the risk of unintended damage to neighboring tissues, thereby enhancing safety. The submuscular implant-based technique significantly lowers the likelihood of complications, such as fluid accumulation from muscle fiber dissection or implant exposure/extrusion [5]. However, in lean individuals, there may be instances where implant edges become exposed or palpable. Additionally, some patients seek to augment the width of their hips and increase their hip-to-waist ratio while still preserving the projection that comes with gluteal implants. In such cases, the inclusion of fat grafting proves to be an effective treatment option. It is crucial, however, to perform the injection subcutaneously only, as intramuscular injections have been associated with potentially life-threatening complications [6,7,16]. Therefore, the injection process needs to be performed with the use of intraoperative ultrasound imaging. This can ensure that the injection is performed into the subcutaneous plane. Obtaining satisfactory results with our technique relies on thorough patient selection; lean patients with limited fat for the entire buttocks but sufficient fat for fat grafting to the lateral buttocks and hips are the most suitable for this operation.

There is a significant lack of comprehensive studies addressing gluteal augmentation through the use of fat grafts and implants [1,17,18]. The combination of fat grafting and buttock implants has been described as a primary and secondary technique for gluteal augmentation. The secondary technique, which involves liposuctioning and fat grafting after a previous buttocks implant procedure, is primarily employed to address the limitations associated with implant-based gluteal augmentation. These limitations may include visible implant contours, rippling, and palpable implant contours [18]. The combination of implants and fat grafting as the primary method for buttocks augmentation is especially suitable for patients wanting wider hips and those who request an increase in buttocks size greater than what can be obtained with only implants. Studies that have covered this topic in the past have typically employed similar implant pocket locations, primarily intramuscular and subfascial/intramuscular dual-plane [1,17]. These planes for implant placement have been extensively discussed in the literature, and the overall complication rate for gluteal augmentation using silicone implants has been reported to be around 21.6%, with the majority of complications occurring with subfascial implants (62.1%) [3]. In our novel technique, we opted for submuscular implant placement. This choice has been associated with lower complication rates than other planes [5]. However, it is important to note that the complication rate is still higher than in gluteal augmentation through liposuction and fat grafting [3].

In studies covering the combination of fat grafting with implants, the overall complication rate has ranged from 7.4% to 15.9% [1,11,17,18]. In our current study, the complication rate was 6.0%, with the majority of complications being linked to the implants. Fortunately, all complications were successfully treated, and no secondary procedures were needed. Post-operative surgical site infections after gluteal augmentation with implants are a more frequent complication compared to other aesthetic surgeries [19,20]. This is mainly related to the close proximity of the anus to the surgical incision. Usually, the cases of surgical site infections are relatively easy to treat, necessitating antibiotic treatment [5]. However, these infections may become severe, even leading to cases of necrotizing fasciitis, as shown in the case report presented by Stojičić et al. [21]. Our study demonstrates that the combination of both submuscular implants and fat grafting did not increase the infection rates. The relatively low frequency of complications observed in the present study may be associated with the addition of ultrasound guidance during the fat grafting step. Fat injection to the gluteal region has been associated with potentially fatal complications, namely the formation of pulmonary fat embolisms. In a recent review where 25 fatal cases of macroscopic fat embolism in South Florida were analyzed, it was concluded that intramuscular fat injections were responsible for the formation of macroscopic fat embolisms [8,22]. Traditionally, the fat grafting step was performed blindly, with the surgeons identifying the correct location of the cannula based solely on feel and experience [8]. However, due to the increased mortality associated with fat grafting in the gluteal region, many techniques and recommendations have been proposed in order to decrease the risk of intramuscular injections [23,24,25]. Regrettably, there have been multiple instances of macroscopic fat embolism-caused deaths where experienced surgeons only noted subcutaneous injection, yet postmortem examinations revealed evidence of fat infiltration into the muscular plane [22]. This demonstrates that the tactile perception of where the injection occurs, along with the experience of the surgeon, is not enough to decrease the risk of this fatal complication. In our opinion, to decrease the risks associated with fat grafting, the injection process should be performed under ultrasound guidance. Nonetheless, adequate knowledge and experience with gluteal augmentation with fat grafting and implants is still of utmost importance in order to perform this procedure safely and provide patients with satisfactory results.

**Figure 3:**
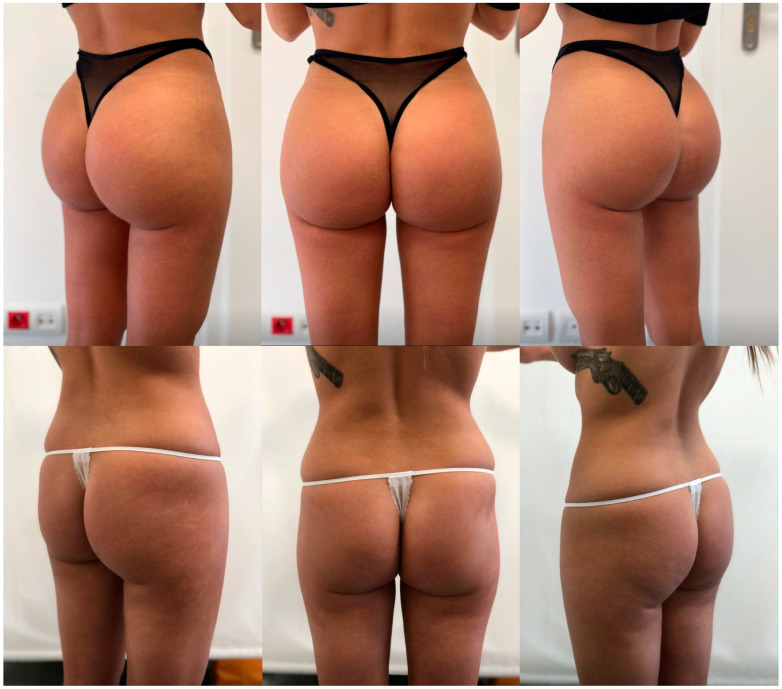
Results in a 28-year-old female patient 24 months after surgery (implant size: Polytech 390; fat graft: 350 mL on each side; height: 162 cm; weight: 57 kg). A tattoo was removed from the patient’s left scapula.

**Figure 4:**
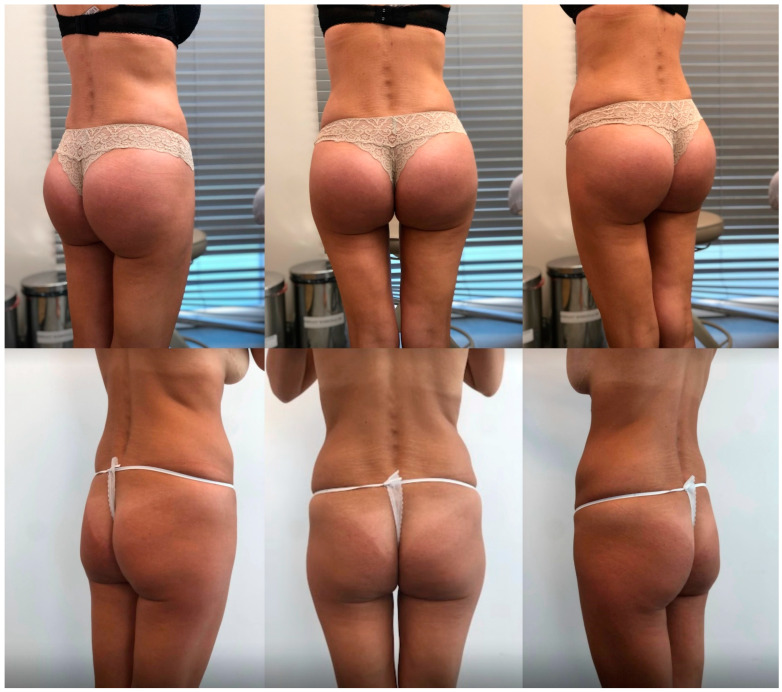
Results in a 43-year-old female patient 24 months after surgery (implant size: Polytech 340; fat graft: 300 mL on each side; height: 166 cm; weight: 62 kg).

**Figure 5:**
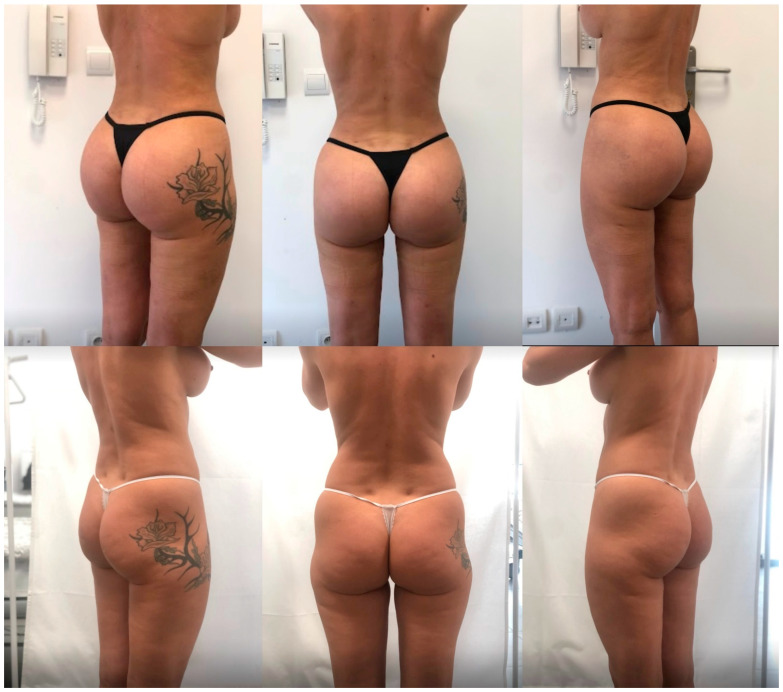
Results in a 34-year-old female patient 26 months after surgery (implant size: Polytech 390; fat graft: 350 mL on each side; height: 175 cm; weight: 64 kg).

Overall, satisfaction levels achieved through this innovative technique were the highest observed in our center, surpassing those from isolated implant-based augmentation and buttocks contouring with liposuctioning and fat grafting (Table 2, Figure 3, Figure 4 and Figure 5). We strongly believe that the presented technique delivers reliable aesthetic results with adequate safety.

A notable limitation in the present study is the sample size; a larger cohort of patients would be required to ascertain this technique’s benefits and risks fully. Furthermore, we did not conduct a structured and standardized evaluation of aesthetic outcomes using a certified scale, making it challenging to measure and compare the aesthetic results among patients quantitatively. Patient satisfaction was not studied using a standardized questionnaire, which may be another source of potential bias.

## 5. Conclusions

In conclusion, our method for gluteal augmentation, utilizing ultrasonic liposuction, submuscular silicone implants, and ultrasound-guided fat grafting to the buttocks and/or hips, represents a safe and effective approach to enhancing buttock aesthetics. Particularly beneficial for patients desiring augmentation in both central and lateral regions of the buttocks, especially when lacking ample fat tissue for traditional fat grafting methods, this approach offers a secure solution with satisfying outcomes. The addition of ultrasound imaging while performing the fat injection step increases the safety of the procedure.

## Figures and Tables

**Table 1:** General characteristics of the 50 female patients included in the present study. IQR—Interquartile Range; kg—Kilograms; cm—Centimeters; BMI—Body Mass Index; *—Division into cohorts was based on BMI: (1) Underweight: BMI < 18.5; (2) Normal Weight: BMI ≤ 18.5–25.0; (3) Overweight: BMI ≤ 25.0–30.0; (4) Obese: BMI ≤ 30.0–34.9.

Group	Category	N	Mean	Standard Deviation	Median	Min	Max	IQR
Overall	Age	50	33.90	6.83	33.00	21.00	52.00	8.50
	Weight [kg]		59.23	8.52	58.00	47.50	101.00	8.50
	Height [cm]		167.44	6.38	168.00	155.00	185.00	7.50
	BMI		21.08	2.27	20.82	17.38	30.83	2.24
Underweight *	Age	5	31.80	8.47	35.00	23.00	42.00	36.00
	Weight [kg]		51.54	4.31	50.00	47.90	59.00	50.80
	Height [cm]		169.10	6.29	166.00	165.00	180.00	169.00
	BMI		18.00	0.41	18.21	17.38	18.37	18.25
Normal Weight *	Age	44	33.93	6.78	33.00	21.00	52.00	37.25
	Weight [kg]		59.02	5.87	58.50	47.50	75.00	63.00
	Height [cm]		166.82	6.15	167.00	155.00	185.00	170.25
	BMI		21.20	1.62	20.98	18.59	24.92	22.18
Obese *	Age	1	37.00					
	Weight [kg]		101.00					
	Height [cm]		181.00					
	BMI		30.83					

**Table 2:** Results regarding the patients’ satisfaction survey. *—Division into cohorts was based on BMI: (1) Underweight: BMI < 18.5; (2) Normal Weight: BMI ≤ 18.5–25.0; (3) Overweight: BMI ≤ 25.0–30.0; (4) Obese: BMI ≤ 30.0–34.9.

Group	Category	Very Satisfied	Rather Satisfied	Rather Dissatisfied	Very Dissatisfied
Overall (N = 50)	The shape of the buttocks	43 (86.0%)	4 (8.0%)	3 (6.0%)	0 (0.0%)
	The projection of the buttocks	42 (84.0%)	8 (16.0%)	0 (0.0%)	0 (0.0%)
	The size of the buttocks	45 (90.0%)	4 (8.0%)	1 (2.0%)	0 (0.0%)
	The general silhouette shape	44 (88.0%)	6 (12.0%)	0 (0.0%)	0 (0.0%)
	The shape of the abdomen and waist	42 (84.0%)	8 (16.0%)	0 (0.0%)	0 (0.0%)
Underweight * (N = 5)	The shape of the buttocks	3 (60.00%)	2 (40.00%)	0 (0.00%)	0 (0.00%)
	The projection of the buttocks	4 (80.00%)	1 (20.00%)	0 (0.00%)	0 (0.00%)
	The size of the buttocks	4 (80.00%)	1 (20.00%)	0 (0.00%)	0 (0.00%)
	The general silhouette shape	4 (80.00%)	1 (20.00%)	0 (0.00%)	0 (0.00%)
	The shape of the abdomen and waist	5 (100.00%)	0 (0.00%)	0 (0.00%)	0 (0.00%)
Normal Weight * (N = 44)	The shape of the buttocks	39 (88.64%)	2 (4.55%)	3 (6.82%)	0 (0.00%)
	The projection of the buttocks	38 (86.36%)	6 (13.64%)	0 (0.00%)	0 (0.00%)
	The size of the buttocks	40 (90.91%)	3 (6.82%)	1 (2.27%)	0 (0.00%)
	The general silhouette shape	40 (90.91%)	4 (9.09%)	0 (0.00%)	0 (0.00%)
	The shape of the abdomen and waist	36 (81.82%)	8 (18.18%)	0 (0.00%)	0 (0.00%)
Obese * (N = 1)	The shape of the buttocks	1 (100.00%)	0 (0.00%)	0 (0.00%)	0 (0.00%)
	The projection of the buttocks	0 (0.00%)	1 (100.00%)	0 (0.00%)	0 (0.00%)
	The size of the buttocks	1 (100.00%)	0 (0.00%)	0 (0.00%)	0 (0.00%)
	The general silhouette shape	0 (0.00%)	1 (100.00%)	0 (0.00%)	0 (0.00%)
	The shape of the abdomen and waist	1 (100.00%)	0 (0.00%)	0 (0.00%)	0 (0.00%)

**Table 3:** Distribution of the implants used in the 50 consecutive female patients. *—Division into cohorts was based on BMI: (1) Underweight: BMI < 18.5; (2) Normal Weight: BMI ≤ 18.5–25.0; (3) Overweight: BMI ≤ 25.0–30.0; (4) Obese: BMI ≤ 30.0–34.9.

Group	Implant Type	Total	285	340	360	390	400	440	560
Overall	Polytech	44	1	14	-	25	-	-	4
	Sebbin	6	-	-	2	-	2	2	-
Underweight * (N = 5)	Polytech	5	1	2	-	2	-	-	-
	Sebbin	0	-	-	-	-	-	-	-
Normal Weight * (N = 44)	Polytech	39	-	12	-	23	-	-	4
	Sebbin	5	-	-	2	-	2	1	-
Obese * (N = 1)	Polytech	0	-	-	-	-	-	-	-
	Sebbin	1	-	-	-	-	-	1	-

## Data Availability

The data that support the findings of this study are available from the corresponding author, upon reasonable request.

## Supplementary Materials

The following supporting information can be downloaded at: https://www.mdpi.com/article/10.3390/jcm13102856/s1, Video S1: Video illustrating our hybrid technique for gluteal augmentation.
